# Relationship of hyposalivation and xerostomia in Mexican elderly with socioeconomic, sociodemographic and dental factors

**DOI:** 10.1038/srep40686

**Published:** 2017-01-17

**Authors:** Horacio Islas-Granillo, Aida Borges-Yáñez, Miguel Ángel Fernández-Barrera, Leticia Ávila-Burgos, Nuria Patiño-Marín, María de Lourdes Márquez-Corona, Martha Mendoza-Rodríguez, Carlo Eduardo Medina-Solís

**Affiliations:** 1 Academic Area of Dentistry of Health Sciences Institute at Autonomous University of Hidalgo State, Pachuca, Mexico; 2 DEPeI Faculty of Dentistry of National Autonomous University of Mexico, Ciudad de Mexico, Mexico; 3 Health Systems Research Centre at National Institute of Public Health, Cuernavaca, Mexico; 4 Clinical Research Laboratory of Dental Sciences Doctorate Program at Autonomous University of San Luis Potosí, San Luis Potosí, Mexico; 5 Advanced Studies and Research Center in Dentistry “Dr. Keisaburo Miyata” of School of Dentistry at Autonomous University State of Mexico, Toluca, México

## Abstract

We determined the prevalence of hyposalivation and xerostomia in older Mexicans (≥60 years), and its relationship with diverse factors. A cross-sectional study was realized in elderly subjects from Pachuca, Mexico. Chewing-stimulated saliva was collected under standardized conditions and salivary flow was measured; subjects were considered to have hyposalivation if their stimulated salivary flow was less than 0.7 mL per minute. Xerostomia was evaluated by asking subjects ‘Does your mouth feel dry?’. Hyposalivation was present in 59.7%, and xerostomia in 25.2% of subjects. 16.5% of subjects had both conditions. Xerostomia was present in 27.7% of subjects with hyposalivation and 21.4% of subjects without hyposalivation, but the difference was not significant (p > 0.05). Thus, 68.3% of older Mexicans had xerostomia and/or hyposalivation. Factors associated with hyposalivation were: using fewer devices in oral hygiene, lacking social benefits for retirement/pension, living in a public retirement home, brushing teeth less than twice a day and lacking teeth without dentures. None of the factors included in this study were associated with xerostomia. We concluded that several variables studied were associated with hyposalivation, but none for xerostomia. Additional research should examine the amount of hyposalivation and factors associated with hyposalivation especially in elderly with increased risk for hyposalivation.

Healthy humans produce 0.5–1.5 liters of saliva each day. About 90% of the saliva is derived from three pairs of major salivary glands (parotid, submandibular and sublingual) and the remaining 10% comes from numerous minor salivary glands distributed in the oral mucosa^1^. Saliva is vital for the maintenance of normal oral physiology and mucosal and dental health, and is of paramount importance for the maintenance of oral and general homeostasis. Saliva plays a crucial role in digestive function, speaking, chewing, swallowing, tasting, phonation, cleaning, hydration of the oral mucosa and protection of the teeth, due to buffering and remineralization properties. In addition, saliva controls the composition of the oral microflora due to antibacterial, antifungal and antiviral properties. The loss of salivary function (“dry mouth”) can have far-reaching consequences, such as increased buccodental disease (including dental caries, periodontal disease, gingivitis, erosion and ulceration of mucosal tissues, mucositis and angular cheilitis, and oral candidiasis), speech impairment, denture wearing, less enjoyment and ingestion of food and decreased quality-of-life^2^,^3^,^4^,^5^,^6^,^7^. Dry mouth is most commonly caused by alterations in salivary gland function, dehydration, and cognitive alterations in older people. Salivary dysfunctions can be divided into three alterations: xerostomia (subjective alteration), hyposalivation (reduction of salivary flow), and alterations in salivary composition. Most authors agree that xerostomia and hyposalivation are two separate entities: xerostomia denotes the subjective feeling, the symptom, of dry mouth, whereas hyposalivation denotes the sign, a decreased saliva flow rate^3^,^8^. The term “dry mouth” has been used to describe both conditions^9^.

The diagnosis of salivary dysfunctions can be obtained by means of subjective and objective methods. Xerostomia is primarily evaluated through the use of questionnaires, using either single-item approaches or multi-item scales. Several authors have suggested using instruments to broaden the analysis of xerostomia, in grading aspects related to chewing, swallowing, speech, sleep and quality-of-life. Although a consensus has not been reached on the definition of low salivary flow^7^,^10^, hyposalivation is considered when the salivary flow rate is <0.1 mL/min at rest or <0.7 mL/min under stimulation. Salivary flow rates are measured by sialometry, which is a technique that collects whole saliva or the fluid produced by each major gland individually, either at rest or during stimulation^3^.

The exact nature of the relationship between xerostomia and hyposalivation in the elderly is unknown^11^. Not all people who have hyposalivation report xerostomia, and people who report xerostomia may have normal or high salivary flow. Mouth dryness is a common clinical complaint in the elderly, more so than in other age groups. It is important to comprehensively establish the current status of xerostomia and hyposalivation in the elderly. However, only a few studies have investigated the prevalence of both xerostomia and hyposalivation within the same elderly populations^6^. A recent review by Liu *et al*.^9^ indicated that the prevalence of xerostomia ranged from 5.5% to 39% in the general public and from 17% to 40% among community-dwelling elders. In institutionalized elders, the prevalence of xerostomia ranged from 20% to 72%. The prevalence of hyposalivation ranged from 15% to 23% in community-dwelling elders and from 17% to 50% in institutionalized elders. Some researchers found that unstimulated whole saliva indicated a higher prevalence of hyposalivation than did stimulated whole saliva, whereas other researchers found the opposite results.

Many factors have been identified as possible causes of reduced salivary flow rates: medications, systemic diseases, aging^12^,^13^, the female gender^6^,^11^,^12^,^13^, agents affecting digestive organs^6^, nutritional or general health status, diagnosed diseases^13^,^14^, depression^14^, body mass index^13^, the number of remaining teeth, having fewer than 20 teeth^13^, periodontal condition, xerogenic drugs^13^, consumption of certain nutrients^14^, and bite force^4^. Variables associated with xerostomia include sex^11^,^15^, age^15^, various diseases (such as diabetes, hypertension, cardiovascular disorders, neurological disorders and psychological disorders)^15^,^16^, various drugs^15^, hypnotics^6^, smoking habits^6^ and depression^6^.

To our knowledge, no studies have identified risk factors for reduced salivary flow rate and xerostomia in the Mexican elderly. The aim of this study was to determine the prevalence of hyposalivation and xerostomia in a sample of older Mexicans, and to evaluate its association with sociodemographic, socioeconomic, behavioral and dental variables.

## Materials and Methods

### Study design and sample

A study was conducted in subjects ≥60 years-old recruited from two retirement homes (one public and one private) and one group of community-dwelling, independently-living people. The group meets three days a week in the city of Pachuca, in the Hidalgo province of Mexico, for recreation, culture and entertainment. The study design and subjects have been described in detail^17^,^18^,^19^,^20^. Briefly, a cross-sectional study was conducted. Nowadays, in the Hidalgo province are living 250,715 people with 60 years or older; only In Pachuca City about 23, 340 elder people are living. Otherwise in this City there are eight homes for elderly, but only three with different type of subsidy were chosen for this study. After obtaining the relevant permits, we invited the subjects to participate in the study, informing them about the aims of the research, the confidentiality in data management, and the fact that they could stop participating at any moment. Inclusion criteria were: 1) either sex, 2) 60 years and older, 3) a wish to participate in the research, and 4) coming from the above-mentioned groups. Exclusion criteria were: 1) under 60 years-old, 2) having a hearing or language impairment that could affect the interview, and 3) having a physical or mental disability that could preclude the oral exam. We did not use any sampling approach. From the initial sample of 151 subjects, 12 refused to participate in the study or did not meet the inclusion/exclusion criteria, for that reason, in the final sample just participated 139 subjects.

### Variables and data collection

The dependent variables used in this analysis were hyposalivation and xerostomia. To determine stimulated salivary flow, standardized procedures were applied^21^. Stimulated saliva samples were taken under resting conditions in a quiet room between 7:30 a.m. and 9:30 a.m., at least one hour after the last intake of food and beverages or smoking too, for this study, tooth brushing and rinsing were not allowed; because these variables could modify the salivary pH grade. The subjects chewed a piece of paraffin wax of fixed size (Merck 7159) for five minutes, during which the whole saliva was swallowed and expectorated into a calibrated dry plastic tube. The flow rate was calculated in milliliters per minute (mL/min). When the stimulated salivary flow was lower than 0.7 mL/min, the subjects were considered to exhibit hyposalivation. In addition, the subjects were evaluated for xerostomia with the question ‘Does your mouth feel dry?^22^,^23^ and were operationally categorized as 0 (subjects without xerostomia, who answered “no” to the question) and 1 (subjects with xerostomia, who responded “yes” to the question).

Questionnaires were used to collect information on demographic variables such as age, sex and marital status, as well as indicators of socioeconomic position such as home type, social security, pension/retirement and education. Questionnaires also addressed various other exposures and behavioral factors such as the frequency of tooth brushing, use of aids in oral hygiene, smoking, consumption of soda, number of drugs, number of hyposalivation-inducing medications, presence of chronic diseases and whether the subject had received radiation in the head or neck. A thorough clinical examination by one examiner was used to determine the number of missing teeth and use of dentures. In this regard, the subjects were coded as 0 (subjects who had lost all of their teeth (edentulous) and used dentures), 1 (subjects who had lost at least one tooth and used fixed or removable prostheses), 2 (subjects who were edentulous and did not use dentures), 3 (subjects with at least 20 natural teeth and no prostheses), and 4 (subjects who had less than 20 natural teeth and no prostheses).

### Data analysis

Statistical analysis was performed using Stata statistical package version 11.0 (StataCorp, Texas, United States). Subjects’ characteristics were summarized as means (±standard deviation) and range (minimum to maximum) for continuous variables, as well as frequency counts and percentages for categorical data. Subsequently, a bivariate analysis was performed; chi-squared and Fisher’s exact tests were used to analyze categorical variables, and the Mann-Whitney test was used to compare quantitative data between the groups. P values <0.05 were considered to be statistically significant.

### Ethical considerations

The study protocol followed the Declaration of Helsinki for research involving human subjects and was approved by the local ethics committee of the Post Graduate and Research Unit of the Academic Area of Dentistry of Health Sciences Institute at Autonomous University of Hidalgo State. Written informed consent was obtained from all participants who agreed to participate in this study as volunteers.

## Results

In total, the study included 139 adults over 60 years of age. The characteristics of the subjects are shown in Table 1. The average age was 79.06 ± 9.78 years, and 69% of the subjects were female. The prevalence of hyposalivation was 59.7% (95% CI: 51.5–68.0), whereas the prevalence of xerostomia was 25.2% (95%CI: 17.9–32.5) (Table 2). The salivary flow rate of the whole study group was 0.75 ± 0.80 mL/min. Additionally, 16.5% of the subjects had both hyposalivation and xerostomia. Hence, 68.3% of the subjects in the present study had xerostomia and/or hyposalivation.

The results of the bivariate analysis of hyposalivation can be seen in Table 3 and are related to xerostomia in Table 4. We observed that hyposalivation correlated with using fewer auxiliaries in oral hygiene, lacking social benefits for retirement/pension, living in a public retirement home, brushing teeth less than two times a day, and being edentulous without dentures (p <0.05). None of the variables analyzed in this study correlated with xerostomia. Xerostomia was reported by 27.7% of subjects with hyposalivation and 21.4% of subjects who did not have hyposalivation; this difference was not significant (Table 5).

## Discussion

This cross-sectional study was conducted in elderly Mexican people to investigate the prevalence of hyposalivation and xerostomia, as well as the factors associated with these conditions. Our findings revealed that 59.7% of the subjects had hyposalivation and 25.2% self-reported xerostomia. Liu *et al*.^9^ observed that the prevalence of xerostomia ranged from 17% to 40% among community-dwelling elders and 20% to 72% among institutionalized elders. The prevalence of hyposalivation ranged from 15% to 23% in the community-dwelling elders and 17% to 50% among institutionalized elders. Previous studies suggested that not all people who had hyposalivation reported xerostomia, and people who reported xerostomia could have a normal or high salivary flow^6^,^12^. This was also observed in our study; although subjects with hyposalivation presented a higher prevalence of xerostomia rather than those subjects with lacking of hyposalivation, anyway, the difference was not significant. It has been suggested that hyposalivation can exist in people who have no complaint of xerostomia, while people with sufficient salivary secretion can have xerostomia^6^. However, we measured salivary flow rates during stimulation; studies have shown that oral dryness is more significantly associated with the resting salivary flow rate than the stimulated salivary flow rate^13^,^14^.

In recent decades, studies have analyzed the impact of socioeconomic position on the general health of the population. Health is, to a large extent, determined by social class and socioeconomic position, referred to as health inequalities. This health stratification produces differences in outcomes of morbidity and mortality, depending on which stratification measure is used. The exact mechanism by which health and socioeconomic status are associated is unclear, because this variable is part of a multidimensional construct^24^. Possible mechanisms between socioeconomic position and differences in health involve health behaviors, affordability of a healthy lifestyle and healthcare, and absence of physically demanding work and hazardous working conditions or distress. Indeed, the associations of socioeconomic position and health are so strong that several researchers have put forward the hypothesis that social standing may indeed be the fundamental cause for health^25^. Krieger^26^ introduced the concept of “biological expression of social inequality”, which refers to how people biologically incorporate and express their experiences of economic and social inequality, from *in utero* to death, thereby manifesting social inequalities across a wide range of health aspects. Another hypothesis to explain this association is that hyposalivation may fit into the ‘Life course perspective’^27^. According to this concept, during growth the individual passes through critical periods when socioeconomic and biological factors may affect the development of organs and body tissues. Deficiencies in certain factors may lead to a permanently reduced function of the salivary glands and to an increased susceptibility to disease during adulthood^13^. In fact, various studies worldwide have found that the best socioeconomic position is associated with better oral health events^28^,^29^,^30^,^31^. We analyzed diverse indicators of socioeconomic position and found two of them to be significant: having a pension/retirement and living in a private retirement home or and one group of community-dwelling (the club of day). Similar to our study, a study in Japan found that subjects with hyposalivation were more likely to have a lower socioeconomic position^14^. In contrast, a study in Brazil did not obtain the same results^32^.

In general, daily tooth brushing has a positive effect on oral health by preventing major oral diseases, Studies have shown that daily tooth brushing also has a beneficial impact on salivary flow, which our results support. In the Netherlands, Ligtenberg *et al*.^33^ found that tooth brushing induced transient changes in salivary flow. After brushing with water, the subjects’ salivary secretion rate increased significantly for 60 minutes, suggesting that tooth brushing mechanically stimulates saliva secretion. The secretion rates were further enhanced after brushing with different types of toothpastes, probably as a result of additional gustatory stimulation. A study conducted in Japan by Inenaga *et al*.^34^ found that tooth brushing in different oral regions (surface of the molars, gingiva, tongue, and palatal rugae) increased the flow rate of the parotid salivary gland, probably via the activation of periodontal mechanoreceptors. Hoek *et al*.^35^ found that tooth brushing increased the saliva flow rate by 15%, but the flow rate returned to baseline within 30 minutes; thus, the brushing of teeth induces a transient increase in saliva secretion. Since no toothpaste was used, the increase was probably due to mechanical, not gustatory, stimulation. Similarly, other studies have shown that brushing teeth with a manual or electric toothbrush significantly improves the feeling of xerostomia and the amount of salivary flow. Mechanical and electrical stimulation have been used in the past to increase salivary flow. Sonic vibration stimulation with an electric toothbrush is another useful way to stimulate saliva secretion^36^.

The association between hyposalivation and the number of teeth in the mouth has been observed in various studies around the world. In Sweden, Flink *et al*.^13^ found that subjects with less than 20 teeth had a higher risk of hyposalivation (<0.70 mL/min). Studies by Samnieng *et al*.^37^ (community-dwelling elderly Thai) and Sawair *et al*.^38^. (Jordanian Arabs 15 years and older) found that subjects with hyposalivation had fewer teeth than control subjects. Our results are consistent with both of these studies. Bite force is a key determinant of masticatory performance, and correlates with chewing efficiency in subjects with overdentures, full dentures, and natural dentitions. Bite force is involved in the mastication of food, and the force required to chew food is related to the firmness of one’s diet. An increase in the frequency of chewing or a change in diet to more rigid foods results in increased salivary flow rates^39^. Although not explicitly studied, we observed that when missing teeth were replaced with prostheses, the salivary flow was higher. It has been suggested that both chewing and bite force are involved in salivary gland secretion^39^; multiple authors consider the loss of teeth to be responsible for the decrease in bite force and subsequent decrease in salivary flow rate. Matzuda *et al*.^40^ found that the replacement of complete dentures for elderly patients improved maximal occlusal force and increased both the stimulated and unstimulated salivary flow rates; consequently, the number of subjects with hyposalivation decreased significantly. Prosthetic treatment providing more functional complete dentures may increase salivary flow by allowing more frequent or forceful mastication and stimulation of mechanoreceptors in the oral mucosa. An increase in salivary flow rate accompanying the insertion of an initial set of complete dentures is a well-known phenomenon^36^. Our study suggests that there is an increased stimulation in subjects with more natural or prosthetic teeth. In any regard, our results reinforce the need to rehabilitate individuals with missing teeth and hyposalivation to improve oral health and quality-of-life.

Studies have investigated the effects of aging on salivary gland secretion, but these effects remain unclear. Many functional studies found that there is no decrease in the flow of whole or parotid saliva with age in healthy, non-medicated individuals^1^. The results of our study reinforce this notion because we did not observe a significant association between age and xerostomia or hyposalivation. However, we used only elderly subjects, so younger individuals are needed to verify the hypothesis. In our study, none of the independent variables correlated with xerostomia, as seen in other studies.

The limitations of this study should be recognized for a correct interpretation of the results. We used a cross-sectional study design, which provides only statistical associations and cannot establish causal relationships between the variables. In the other hand, in this study we only measured stimulated salivary flow rate, and other studies demonstrated that the oral dryness is more significantly associated with unstimulated whole saliva flow rate, so, in the future studies this situation should be considered. Finally, nowadays there are other ways to evaluate xerostomia, so, we propose apply this measure system in other questionnaires with more sensibility for xerostomia.

In conclusion, the results show that the prevalences of hyposalivation and xerostomia were 59.7% and 25.2%, respectively. Only 16.5% of the subjects had both hyposalivation and xerostomia. In total, 68.3% of the subjects had xerostomia and/or hyposalivation. Several variables were found to be associated with hyposalivation, but none were associated with xerostomia. These results indicate clear inequalities in hyposalivation by socioeconomic variables. Additional research should examine the prevalence and consequences in high-risk older.

## Additional Information

**How to cite this article**: Islas-Granillo, H. *et al*. Relationship of hyposalivation and xerostomia in Mexican elderly with socioeconomic, sociodemographic and dental factors. *Sci. Rep.* **7**, 40686; doi: 10.1038/srep40686 (2017).

**Publisher's note:** Springer Nature remains neutral with regard to jurisdictional claims in published maps and institutional affiliations.

## Figures and Tables

**Table 1:** Univariate analysis of the characteristics of the subjects included in the study.

	Mean ± sd	Min - Max
Age (years)	79.06 ± 9.78	60–100
Number of aids in oral hygiene	1.47 ± 1.31	0–5
Number of drugs	4.21 ± 2.71	0–9
Number of drugs inducing hyposalivation	3.03 ± 2.18	0–7
	**Frequency**	**Percentage**
Sex		
Male	43	30.9
Female	96	69.1
Health insurance		
Yes	64	46
No	75	53
Pension/retirement		
No benefit	105	75.5
With benefit	34	24.5
Schooling		
Less than High School	110	79.1
High School and more	29	20.9
Type of location		
Publicly funded	84	60.4
Private	31	22.3
Adult day center	24	17.3
Tooth brushing frequency		
Less than twice/day	90	64.7
Two or more times/day	49	35.3
Radiation therapy		
No	133	95.7
Yes	6	4.3
Current tobacco use		
No	117	84.2
Yes	22	15.8
Soft drink intake		
Yes	46	33.1
Sometimes	48	34.5
No	45	32.4
Chronic diseases		
None	37	26.6
Yes	102	73.4
Dental status		
Edentulous with prosthesis	27	19.6
Lost teeth with prosthesis	37	26.8
Edentulous without prosthesis	27	19.6
Lost teeth (<21) without prosthesis	33	23.9
Lost teeth (>20) without prosthesis	14	10.1

**Table 2:** Hyposalivation and xerostomia prevalence in the study.

	Frequency	Percentage	95% CI
Hyposalivation			
No (≥0.7 mL/min)	50	40.3	32.0–48.5
Yes (<0.7 mL/min)	83	59.7	51.5–68.0
Xerostomia			
Absent	104	74.8	67.5–82.1
Present	35	25.2	17.9–32.5

**Table 3:** Bivariate analysis between hyposalivation and the characteristics of the study subjects.

	Hyposalivation		
	Absent	Present	P value
Age (years)	78.86 ± 10.52	79.19 ± 9.31	0.9383
Number of aids in oral hygiene	1.82 ± 1.24	1.23 ± 1.31	**0.0074**
Number of drugs	3.96 ± 2.82	4.37 ± 2.64	0.3584
Number of drugs inducing hyposalivation	2.84 ± 2.16	3.17 ± 2.19	0.3528
Sex			
Male	18 (41.9)	25 (58.1)	0.8
Female	38 (39.6)	58 (60.4)	
Health insurance			
Yes	27 (42.2)	37 (57.8)	0.673
No	29 (38.7)	46 (61.3)	
Pension/retirement			
No benefit	37 (35.2)	68 (64.8)	**0.033**
With benefit	19 (55.9)	15 (44.1)	
Schooling			
Less than High School	20 (40.8)	29 (59.2)	0.925
High School and more	36 (40.0)	54 (60.0)	
Type of location			
Publicly funded	26 (30.9)	58 (69.1)	**0.019**
Private	16 (51.6)	15 (48.4)	
Adult day center	14 (58.3)	10 (41.7)	
Tooth brushing frequency			
Less than twice/day	30 (33.3)	60 (66.7)	**0.023**
Two or more times/day	26 (53.1)	23 (46.9)	
Radiation therapy			
No	53 (39.8)	80 (60.2)	0.62
Yes	3 (50.0)	3 (50.0)	
Current tobacco use			
No	48 (41.0)	69 (59.0)	0.683
Yes	8 (36.4)	14 63.6)	
Soft drink intake			
Yes	18 (39.1)	28 (60.9)	0.968
Sometimes	20 (41.7)	28 (58.3)	
No	18 (40.0)	27 (60.0)	
Chronic diseases			
None	13 (35.1)	24 (64.9)	0.456
Yes	43 (42.2)	59 (57.8)	
Dental status			
Edentulous with prosthesis	12 (44.4)	15 (55.6)	**0.019**
Lost teeth with prosthesis	16 (43.2)	21 (56.8)	
Edentulous without prosthesis	5 (18.5)	22 (81.5)	
Lost teeth (<21) without prosthesis	19 (57.6)	14 (42.4)	
Lost teeth (>20) without prosthesis	3 (21.4)	11 (78.6)	

**Table 4:** Bivariate analysis between xerostomia and the characteristics of the study subjects.

	Xerostomia		
	Absent	Present	P value
Age (years)	78.99 ± 9.73	79.26 ± 10.04	0.8251
Number of aids in oral hygiene	1.46 ± 1.28	1.48 ± 1.42	0.9738
Number of drugs	4.18 ± 2.73	4.28 ± 2.71	0.8547
Number of drugs inducing hyposalivation	3.01 ± 2.18	3.11 ± 2.21	0.916
Sex			
Male	32 (74.4)	11 (25.6)	0.942
Female	72 (75.0)	24 (25.0)	
Health insurance			
Yes	47 (73.4)	17 (26.6)	0.729
No	57 (76.0)	18 (24.0)	
Pension/retirement			
No benefit	78 (74.3)	27 (25.7)	0.799
With benefit	26 (76.5)	8 (23.5)	
Schooling			
Less than High School	36 (73.5)	13 (26.5)	0.787
High School and more	68 (75.6)	22 (24.4)	
Type of location			
Publicly funded	66 (78.6)	18 (21.4)	0.144
Private	19 (61.3)	12 (38.7)	
Adult day center	19 (79.2)	5 (20.8)	
Tooth brushing frequency			
Less than twice/day	68 (75.6)	22 (24.4)	0.787
Two or more times/day	36 (73.5)	13 (26.5)	
Radiation therapy			
No	100 (75.2)	33 (24.8)	0.638
Yes	4 (66.7)	2 (33.3)	
Current tobacco use			
No	86 (73.5)	31 (26.5)	0.41
Yes	18 (81.8)	4 (18.2)	
Soft drink intake			
Yes	38 (82.6)	8 (17.4)	0.299
Sometimes	35 (72.9)	13 (27.1)	
No	31 (68.9)	14 (31.1)	
Chronic diseases			
None	28 (75.7)	9 (24.3)	0.889
Yes	76 (74.5)	26 (25.5)	
Dental status			
Edentulous with prosthesis	20 (74.1)	7 (25.9)	0.272
Lost teeth with prosthesis	31 (83.8)	6 (16.2)	
Edentulous without prosthesis	16 (59.3)	11 (40.7)	
Lost teeth (<21) without prosthesis	25 (75.8)	8 (24.2)	
Lost teeth (>20) without prosthesis	11 (78.6)	3 (21.4)	

**Table 5:** Analysis between hyposalivation and xerostomia.

	Xerostomia		
	Absent	Present	P value
Hyposalivation			
Absent	44 (78.6)	12 (21.4)	X^2^ = 0.7005
Present	60 (72.3)	23 (27.7)	0.403
